# Independent variables of pH: Ten Knights of the Hydrogen Ion Kingdom-Part I. A prospective observational study

**DOI:** 10.1371/journal.pone.0306756

**Published:** 2024-07-10

**Authors:** Bulent Gucyetmez, Kaan Gucyetmez, Zeynep Tugce Sarikaya, Lutfi Telci

**Affiliations:** 1 Department of Anesthesiology and Reanimation, Acibadem Mehmet Ali Aydinlar University School of Medicine, Istanbul, Turkey; 2 Department of Computer Science, Carnegie Mellon University, Pittsburgh, PA, United States of America; 3 General Intensive Care Unit, Acibadem International Hospital, Istanbul, Turkey; University of Health Sciences, Beyhekim Training and Research Hospital, TÜRKIYE

## Abstract

CO_2_, HCO_3_, SID_,_ and total weak acids have been defined as pH’s independent variables. However, according to Gamble, HCO_3_ should be equal to the difference between the sum of cations and the sum of anions besides HCO_3_. Therefore, if this mathematical expression is substituted for HCO_3_ in the Henderson-Hasselbalch equation, all independent variables of pH can be demonstrated. Our aim is to test this theory in this study. This prospective observational study was conducted between 2019 and 2020. All admitted patients to the intensive care unit who were >18 years old were included. Demographic data, blood gas parameters, albumin, magnesium, and inorganic phosphorus levels, and outcomes were recorded twice (at admission and at the 24^th^ hour). The multivariate linear regression model was used to determine pH’s independent variables. In the multivariate linear regression model, pH was significantly increased by each unit increase in Na, K, Ca, and Mg (mmol L^-1^). In contrast, pH was significantly decreased by each unit increase in CO_2_, Cl, lactate, albumin (g dL^-1^), inorganic phosphorus (mg dL^-1^), and the strong ion gap. Ten independent variables can accurately predict the changes in pH. For this reason, all ten independent variables should be separately evaluated when interpreting the acid-base status. With this understanding, all algorithms regarding acid-base evaluation may become unnecessary.

## Introduction

Independent variables of blood pH must be far more than defined independent variables in time. The essential truth is that hydrogen ion concentration ([H]) is the only determiner of acidity or alkalinization in a solution [1]. Over the years, only HCO_3_ and CO_2_ were used for the acid-base evaluation. From the 1940s to 1970s, many tried to solve the metabolic acid-base abnormalities on the relationships between HCO_3_, CO_2_, the anion gap (AG), the buffer base concept, and standard base excess (SBE) [1–5]. However, the most crucial theory in these years was Gamble’s law of electroneutrality: "The sum of cations equals the sum of anions in the plasma” [6]. In the 1980s, Stewart proposed three independent variables for pH by using the law of electroneutrality: strong ion difference (SID), total weak acids (A_tot_), and CO_2_ [7]. This was the first theory that mentioned independent variables of [H^+^]. In the same decade, corrected AG (AG_corr_) was added to the traditional approach algorithm, and then Fencl derived his algorithm based on the Stewart approach [8, 9]. This millennium, O’Dell and Story used partitioning base-excess models to solve metabolic acid-base disorders by separating the effects of Na-Cl difference, albumin, lactate, and unmeasured anions on the SBE [10, 11]. However, none of these approaches have mentioned all the independent variables of [H^+^]. Yet, according to the Gamble equation, HCO_3_ should be equal to the difference between all cations’ ionic charges (Na, K, Ca, Mg) and all anions’ ionic charges other than HCO_3_ (Cl, albumin, inorganic phosphorus (P_i_)_,_ and unmeasured anions (UA)). In 2005, Schück finally derived the new Henderson-Hasselbalch equation by substituting Stewart’s independent variables for HCO_3_ but there was no mention of the independent variables of [H^+^] in his study [12]. Unexpectedly, he used Stewart’s independent variables and AG_corr_ together to define the metabolic components. In this study, we sought to determine all independent variables of [H^+^] using the Gamble and Henderson-Hasselbalch equations and we hypothesized that there were ten independent variables of pH.

## Materials and methods

### Study design

**Fig 1:**
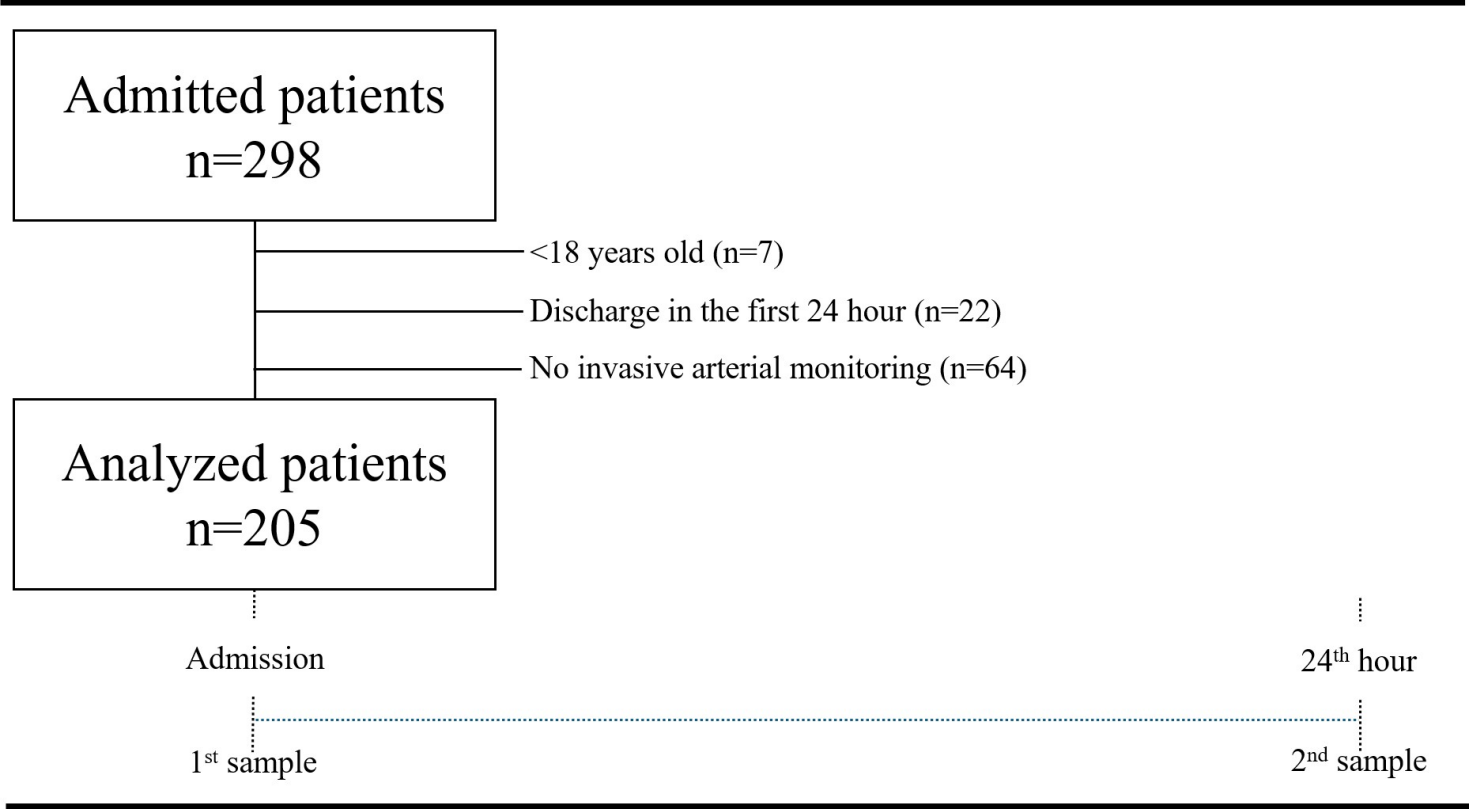
Study flowchart.

This prospective observational study ran from 24 May 2019 to 1 June 2020 after local ethics committee approval (Acıbadem University and Acıbadem Healthcare Institutions Medical Research Ethics Committee -ATADEK- 2019-10/9, Chief: Prof Dr Ismail Hakkı Ulus). All patients admitted to the intensive care unit (ICU) who were >18 years old were included in the study, except that patients without invasive arterial monitoring and whose length of ICU stay (LOS-ICU) was less than 24 hours were excluded (Fig 1) Two arterial blood gas samples (at the moment of ICU admission and the 24^th^ hour) were taken from every patient. Age (years), sex, body mass index (BMI) (kg m^-2^), Charlson comorbidity index (CCI), Acute Physiology and Chronic Health Evaluation II (APACHE II), Sequential Organ Failure Assessment (SOFA) score, pH (temperature-corrected), P_a_CO_2_ (temperature-corrected) (mmHg), HCO_3_, (mmol L^-1^), standard base excess (SBE) (mmol L^-1^), Na (mmol L^-1^), K (mmol L^-1^), Ca (mmol L^-1^), Mg (mg dL^-1^), Cl (mmol L^-1^), lactate (mmol L^-1^), albumin (Alb) (g L^-1^), P_i_ (mg dL^-1^), LOS-ICU and mortality were recorded by an intensivist. The corrected anion gap (AG_corr_) and strong ion gap (SIG) were calculated. Their normal values were defined as 7–17 mmol L^-1^ and 0 mmol L^-1^_,_ respectively [13–15].

### Formulas

1. Mg and P_i_ values were converted from milligrams to millimoles by using the formulas below [16, 17]:
  Mg (mmol L^-1^) = Mg (mg dL^-1^) x 0.41152
  P_i_ (mmol L^-1^) = P_i_ (mg L^-1^) x 0.323
2. [Alb^-^] and [P_i_^-^], A_TOT_, SIG, AG, and AG_corr_ were calculated by using the formulas below [12, 18]:
  [Alb^-^] = ([Alb] (g L^-1^) x [0.123xpH-0.631])
  [P_i_^-^] = ([P_i_] (mmol L^-1^) x [0.309xpH-0.469])
  A_TOT_ = [Alb^-^] + [P_i_^-^]
  SIG = ([Na+K+Ca+Mg]-[Cl+lactate])—(0.0301xP_a_CO_2_x10^(pH-6.1)^)—[Alb^-^]—[P_i_^-^]
  AG = Na+K-Cl-HCO_3_
  AG_corr_ = AG + (0.25 x ([Alb]_normal_-[Alb]_measured_))

Blood samples were taken from the invasive arterial cannula of all patients at two different times: at the ICU admission and the 24^th^ hour of the ICU period. For blood gas samples, syringes with dry heparin were used, and the samples were tested by a blood gas machine in the ICU. As for biochemical parameters, syringes without heparin and tubes were used, and these samples were tested in the biochemistry laboratory. Blood gas data were acquired using an ABL 800 (Radiometer, Denmark, Copenhagen) blood gas device, which employed ion-selective electrodes. Alb, Mg, and P_i_ were acquired using a Cobas C 303 device (Roche, Rotkreuz, Switzerland).

### Theory

1. According to the Henderson-Hasselbalch equation, there are two independent variables of pH:
$$pH=6.1+\log\frac{HCO_{3}}{0.03\times P_{a}CO_{2}}$$ (Eq 1)
2. Gamble JL claimed that the sum of cations equaled the sum of anions in the plasma:[6]
$$Na+K+Ca+Mg=Cl+lactate+HCO_{3}+[Alb^{-}]+[P_{i}^{-}]+[UA]$$ (Eq 2)
3. According to Gamble’s equation, HCO_3_ can be written as below.
$$HCO_{3}=Na+K+Ca+Mg-Cl-lactate-[Alb^{-}]-[P_{i}^{-}]-[UA]$$ (Eq 3)
4. This mathematical expression can be written as a form of the Henderson-Hasselbalch equation instead of in terms of HCO_3_. Since UA’s ionic charge is calculated using SIG [14], the Henderson-Hasselbalch equation can be revised as below:
$$pH=6.1+\log\frac{Na+K+Ca+Mg-Cl-lactate-[Alb^{-}]-[P_{i}^{-}]-[UA]}{0.03\times P_{a}CO_{2}}$$ (Eq 4)
$$pH=6.1+\log\frac{Na+K+Ca+Mg-Cl-lactate-[Alb^{-}]-[P_{i}^{-}]-[SIG]}{0.03\times P_{a}CO_{2}}$$

We tested whether all parameters in the last Henderson-Hasselbalch equation were independent variables of pH.

### Statistical analysis

Descriptive data are presented as mean±sd, median (quartiles), and percentages. The Kolmogorov‒Smirnov test was used to detect normality. Multivariate linear regression models were used to detect independent variables of pH by adding P_a_CO_2_, HCO_3_, Na, Cl, K, Ca, Mg (mmol L^-1^), lactate, Alb (g dL^-1^), P_i_ (mg dL^-1^), and SIG. The Enter method was used to build the model. Furthermore, two multivariate linear regression models were used for the traditional and Stewart approaches. To determine the best model for predicting the pH, leave-one-out cross-validation (LOOCV) scores were used. The correlations and measures of agreements between AG_corr_ and SIG were analyzed with Pearson correlation and the Kappa test, respectively. The estimated power of this study was calculated as 0.99 using the F test for the regression model (effect size f^2^ = 0.35, α = 0.05, total sample size = 410, and the number of predictors = 10, GPower 3.1.9.4). SPSS version 29 was used for all statistical analyses. p<0.05 was accepted as significant.

## Results and discussion

**Fig 2:**
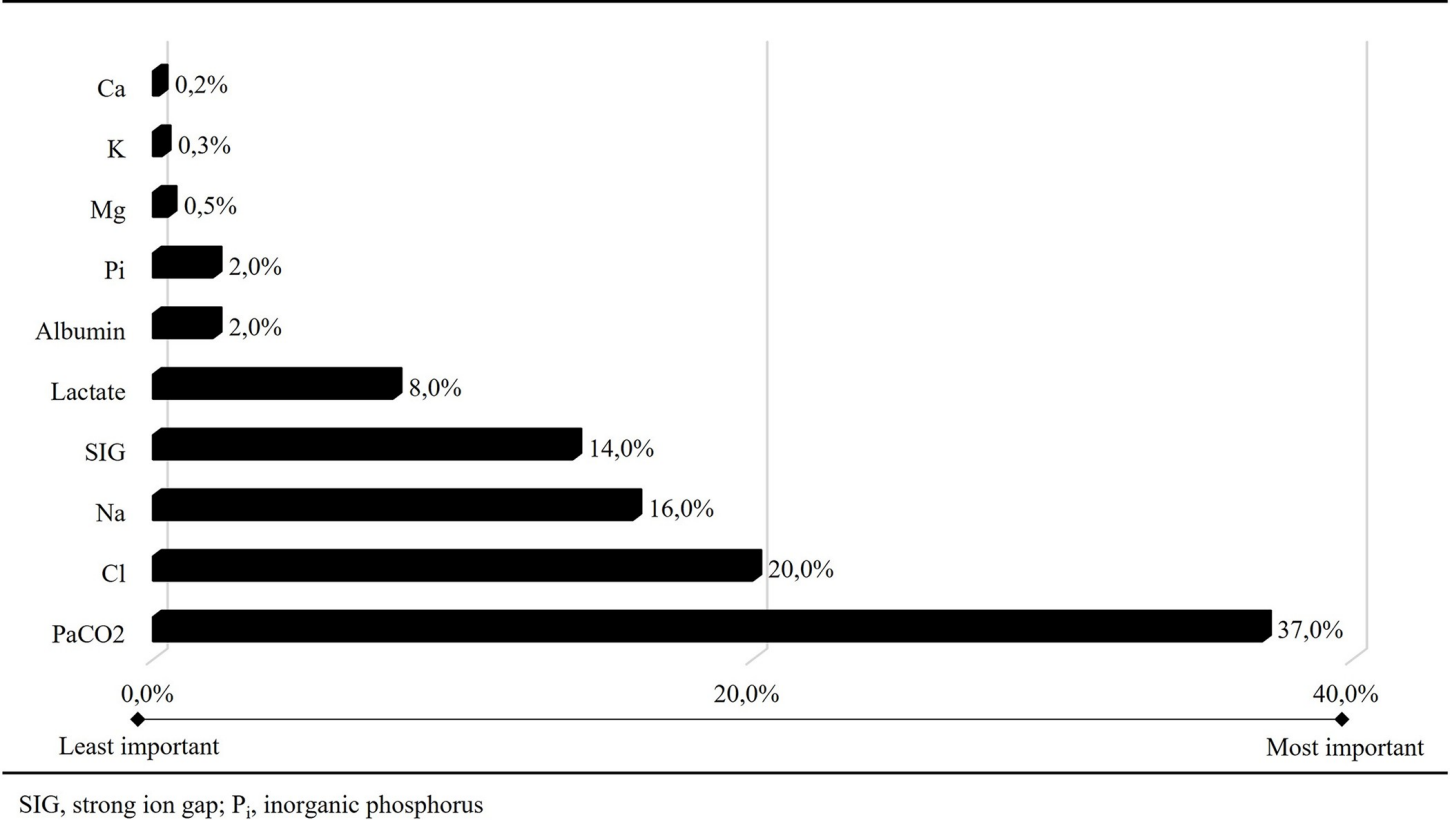
Predictors’ importance on the pH.

**Fig 3:**
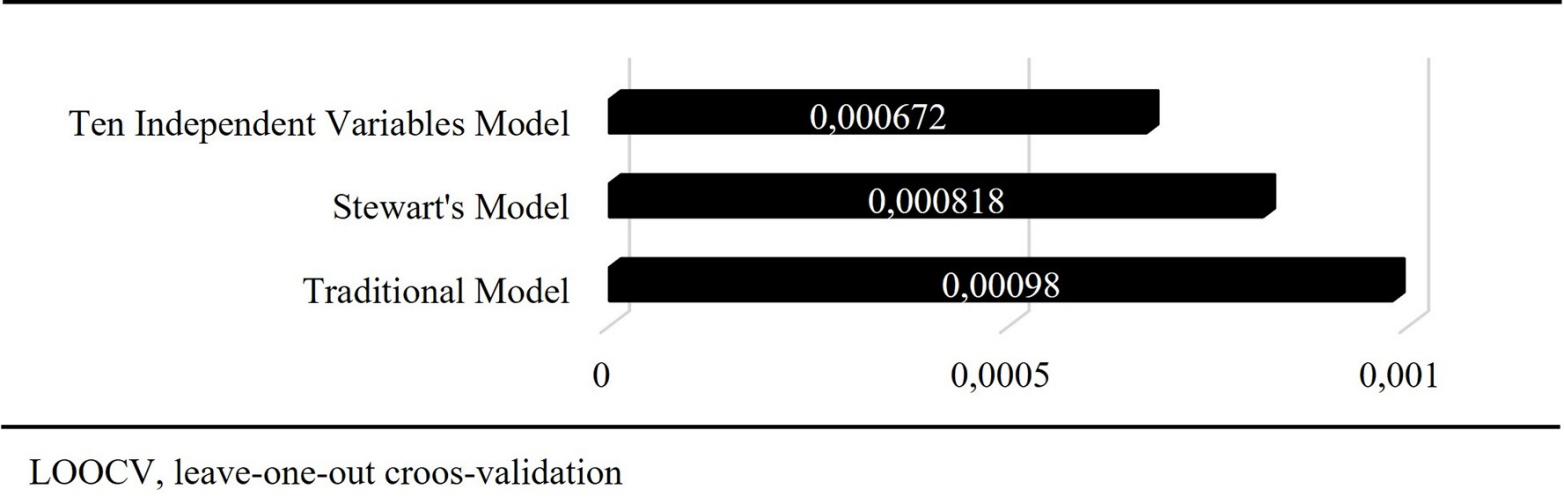
LOOCV scores of three models.

The study evaluated two hundred-five patients with 410 arterial blood gas samples. The median age was 73 years, and the mortality rate was 13.7% (Table 1). For all blood gas samples, the median values of pH, P_a_CO_2_, HCO_3_, Na, Cl, lactate, albumin, P_i,_ and SIG were 7.42, 37.5 mmHg, 24.9 mmol L^-1^, 137 mmol L^-1^, 104 mmol L^-1^, 33 mmol L^-1^, 1.2 mmol L^-1^, 27 g L^-1^, 1.3 mmol L^-1^, and 3.7 mmol L^-1^, respectively (Table 1). In the multivariate linear regression model, pH was 0.015-fold (0.014–0.016), 0.013-fold (0.009–0.017), 0.016-fold (0.002–0.031), and 0.028-fold (0.016–0.040) increased by each unit increase in Na, K, Ca, and Mg (mmol L^-1^), respectively (p<0.001, p<0.001, p = 0.028 and p<0.001, respectively) (Table 2). In contrast, it was 0.009-fold (0.010–0.009), 0.015-fold (0.016–0.014), 0.020-fold (0.021–0.016), 0.005-fold (0.005–0.004), 0.030-fold (0.036–0.025) and 0.016-fold (0.016–0.015) decreased by each unit increase in P_a_CO_2_, Cl, lactate, Alb (g L^-1^), P_i_ (mmol L^-1^), and SIG, respectively (p<0.001, p<0.001, p<0.001, p<0.001, p<0.001, p<0.001, p<0.001 and p<0.001, respectively) (Table 2). For the multivariate linear regression Model III, predictor importance on the pH for P_a_CO_2_, Cl, Na, SIG, lactate, Alb, and P_i_ were 0.38, 0.21, 0.16, 0.14, 0.08, 0.02, and 0.02, respectively (Fig 2). For the traditional model (P_a_CO_2_, HCO_3_, SBE, AG_corr_), Stewart’s model (P_a_CO_2_, SID_a_, A_TOT_, and SIG), and our model (10 independent variables), the adjusted R^2^ values (LOOCV scores) were 0.90 (0.00098), 0.92 (0.00082) and 0.94 (0.00067), respectively (Table 2 and Fig 3). Although there was a positive correlation between AG_corr_ and SIG, their agreement was weak (correlation: 0.76 [0.72–0.80]; kappa coefficient: 0.12) (Table 3).

**Table 1:** Patients’ characteristics, outcomes, and blood gas parameters.

*Patients’ characteristics and outcomes*	
n	205
Age, years	73 (59–83)
Male, n (%)	115 (56.1)
BMI, (kg m^-2^)	26.5 (23.4–30.8)
CCI	6 (4–8)
APACHE II	18 (13–26)
SOFA score	5 (2–7)
Diagnosis, n (%)	
Medical	130 (63.4)
Surgery	69 (33.7)
Emergency surgery	6 (2.9)
Length of ICU stay (min-max)	3 (2–98)
Mortality, n (%)	28 (13.7)
***Blood gas parameters***	
Sample, n	410
pH	7.42 (7.37–7.47)
P_a_CO_2_, (mmHg)	37.5 (32.3–43.7)
HCO_3_, (mmol L^-1^)	24.9 (22.4–27.1)
Na, (mmol L^-1^)	137 (134–140)
Cl, (mmol L^-1^)	104 (100–107)
K, (mmol L^-1^)	3.9 (3.5–4.3)
Ca, (mmol L^-1^)	1.12 (1.06–1.18)
Mg, (mg/dL)	2.1 (1.8–2.4)
Mg, (mmol L^-1^)	0.84 (0.73–1.00)
Lactate, (mmol L^-1^)	1.2 (0.9–1.8)
Albumin, (g L^-1^)	27 (24–31)
[Albumin], (mmol L^-1^)	7.8±1.6
P_i_, (mg dL^-1^)	4.1 (3.2–5.1)
P_i_, (mmol L^-1^)	1.3 (1.1–1.6)
[P_i_], (mmol L^-1^)	2.4 (1.9–3.0)
SIG, (mmol L^-1^)	3.7 (1.7–5.9)

[Albumin]; ionic charge of albumin, APACHE; Acute Physiology and Chronic Health Evaluation, BMI; body mass index, CCI; Charlson comorbidity index, ICU; intensive care unit, [P_i_]; ionic charge of inorganic phosphorus, SBE; standard base excess, SIG; Strong ion gap, SOFA; Sequential Organ Failure Assessment

**Table 2:** Multivariate linear regression models for three approaches.

**Model I; Traditional Approach**		
*Adjusted R*^*2*^: *0*.*90*, *Durbin-Watson*:*2*.*1*, *ANOVA (F = 924 p<0*.*001)*		
	Coefficients (CI 95%)	p
P_a_CO_2_, (mmHg)	-0.008 (-0.008; -0.007)	***<0*.*001***
HCO_3_, (mmol L^-1^)	0.005 (0.000; 0.009)	***0*.*034***
SBE, (mmol L^-1^)	0.010 (0.006; 0.014)	***<0*.*001***
AG_corr_, (mmol L^-1^)	-0.001 (-0.002; 0.000)	***0*.*001***
**Model II; Stewart’s Approach**		
*Adjusted R*^*2*^: *0*.*92*, *Durbin-Watson*:*1*.*73*, *ANOVA (F = 1144 p<0*.*001)*		
	Coefficients (CI 95%)	p
P_a_CO_2_, (mmHg)	-0.010 (-0.011; -0.010)	***<0*.*001***
SID_a_, (mmol L^-1^)	0.017 (0.016; 0.018)	***<0*.*001***
A_TOT_, (mmol L^-1^)	-0.017 (-0.019; -0.015)	***<0*.*001***
SIG, (mmol L^-1^)	-0.017 (-0.018; -0.017)	***<0*.*001***
**Model III; Ten independent variables**		
*Adjusted R*^*2*^: *0*.*94*, *Durbin-Watson*:*1*.*78*, *ANOVA (F = 591 p<0*.*001)*		
	Coefficients (CI 95%)	p
P_a_CO_2_, (mmHg)	-0.009 (-0.010; -0.009)	***<0*.*001***
Na, (mmol L^-1^)	0.015 (0.014; 0.016)	***<0*.*001***
Cl, (mmol L^-1^)	-0.015 (-0.016; -0.014)	***<0*.*001***
K, (mmol L^-1^)	0.013 (0.009; 0.017)	***<0*.*001***
Ca, (mmol L^-1^)	0.016 (0.002–0.031)	***0*.*028***
Mg, (mmol L^-1^)	0.028 (0.016–0.040)	***<0*.*001***
Lactate, (mmol L^-1^)	-0.020 (-0.021; -0.018)	***<0*.*001***
Albumin, (g L^-1^)	-0.005 (-0.005; -0.004)	***<0*.*001***
P_i_, (mg dL^-1^)	-0.030 (-0.036; -0.025)	***<0*.*001***
SIG, (mmol L^-1^)	-0.016 (-0.016; -0.015)	***<0*.*001***

AG_corr_, corrected-anion gap; SBE, standard base-excess; SIG; strong ion gap

**Table 3:** Correlations and measures of agreements between SIG & AG_corr_.

*Correlation*: *0*.*76 (0*.*72–0*.*80)*	AG_corr_>17	7≤AG_corr_≤17	AG_corr_<7	
*Kappa coefficient*: *0*.*12*	(↑UA-Acidosis)	(Normal)	(↑UC-Alkalosis)	Total
SIG				
>0 (↑UA-Acidosis)	148	193	0	341
= 0 (normal)	0	13	0	13
<0 (↑UC-Alkalosis)	2	49	5	56
Total	150	255	5	410

AG_corr_;albumin corrected anion gap, SIG; strong ion gap

This is the first study in which all independent variables of pH were determined empirically. According to our results supported our hypothesis, we detected ten independent variables of pH when considering via the law of electroneutrality: CO_2_, all electrolytes, lactate, albumin, Pi, and UA. Primarily, we believe that two essential facts should be considered: First, according to dissociation theory, the only determiner of acidosis or alkalosis in plasma is [H]. Second, [H] can only be detected by its independent variables. Now, we can discuss all acid-base evaluation approaches based on these facts.

### The traditional approach

This approach is based on the H_2_CO_3_-HCO_3_ buffer system [1, 19, 20]. CO_2_ and HCO_3_ are deemed independent variables. Based on this, acidosis is defined as an increase in CO_2_ or a decrease in HCO_3_, and vice versa for alkalosis [19, 20]. However, CO_2_ is a measured variable, whereas HCO_3_ is a *calculated* variable. Moreover, since HCO_3_ alone is not enough to detect metabolic components, other *calculated* variables, such as SBE and AG, must be added to the evaluation [21]. These *calculated* variables also include other variables in their formulas, including HCO_3_ [21]. In addition, AG should be corrected for albumin at least [22]. To detect whether there are compensation disorders and mixed disorders, rule-of-thumb equations and expected (standard) HCO_3_ are calculated again and assessed; then, a decision about acid-base status is made [19, 21, 23]. For these reasons, the traditional approach has substantial limitations. Firstly, HCO_3_ is a *calculated* variable and cannot be an independent variable for pH. Therefore, other parameters derived from HCO_3_, which are SBE and AG, cannot be independent variables as well. Furthermore, AG should be fully corrected, but if it is fully corrected, it becomes exactly the SIG of Stewart’s approach. For this reason, the measures of agreement between albumin-corrected AG and SIG may be weak (Table 3). Therefore, metabolic acid-base disturbances are tried to be understood by using these *dependent* variables. Secondly, it is perceived as if the H_2_CO_3_-HCO_3_ buffer system is the only system for [H] production. Thus, other probable metabolic variables that affect water dissociation are ignored or overlooked. Further, according to this approach, respiratory disturbance (CO_2_) is compensated for by only metabolic components (HCO_3_) and vice versa [19, 23]. For this reason, compensations or different effects in the metabolic components remain unspecified (e.g., hypochloremic alkalosis and hyperlactatemia under normal CO_2_ levels). Thirdly, algorithms created based on these facts are still complicated and confusing. According to many textbooks, it would seem like there are no acid-base disturbances if pH, CO_2_, and HCO_3_ are normal [15, 24]. We believe that all these reasons may explain why the traditional approach to predicting pH is the weakest (Table 2 and Fig 3).

### Stewart approach and then: New calculated independent variables and corrections

Stewart’s approach creates a solution to metabolic acid-base disturbances by using the electroneutrality law, which Gamble summarizes [6, 7, 18]. In this approach, the independent variables of pH are CO_2_, SID, and A_TOT_. SIG was also added to Stewart’s independent variables [14, 18]. SID, A_TOT_, and SIG are *calculated* variables, whereas CO_2_ is a measured variable in this approach. In this situation, SID and A_TOT_ also relate to their components in both equations [18]. These relationships can cause some problems. For instance, SID can be normal or high in hyperlactatemia, and A_TOT_ can also be normal or low in hyperphosphatemia. To explain these unexpected situations, we must look deeper into Stewart’s equations. Yet, by its definition, an independent variable should have a clear and indisputable effect and thus should not cause these unexpected situations. According to our results, all SID and A_TOT_ components are independent variables of pH (Table 2). Therefore, creating new calculated variables by combining these independent variables is unnecessary. In these circumstances, the only exception is SIG. Interestingly, we found that SIG was an independent variable in this study, although it was a *calculated* variable (Table 2) [14, 18]. Additionally, it was the fourth most important independent variable of pH (Fig 2). Despite these limitations, the prediction of pH using Stewart’s approach is better than using the traditional approach (Table 2 and Fig 3).

In this millennium, the partitioned BE model and chloride corrections have been devised, referring to solving metabolic disturbances [10, 25]. Partitioned BE is a useful method at the bedside, but the relationship of pH with its components has yet to be mentioned. On the other hand, chloride corrections, put forth by Fencl, correct the *observed* serum Cl level with the serum *observed* Na level [9]. If the newly *calculated* Cl level is not between the defined normal limits, it is accepted as hypo- or hyperchloremia. This point of view does not make sense because the observed serum Cl level is *measured* and is the *actual value for serum Cl*, whereas corrections are *calculated* and *imaginary values for serum Cl*. Measured Cl is a real molecule, and this real molecule has a real ionic charge.

### What is the meaning of the existence of ten independent variables?

They have always been there; however, we prefer not to see them as individuals.

This perspective, which is the best at predicting pH (Table 2 and Fig 3), directly links each independent variable to [H]. At this point, it is essential to discuss how and in which direction they affect [H].

### Water resolvents (electrolytes and lactate)

**Fig 4:**
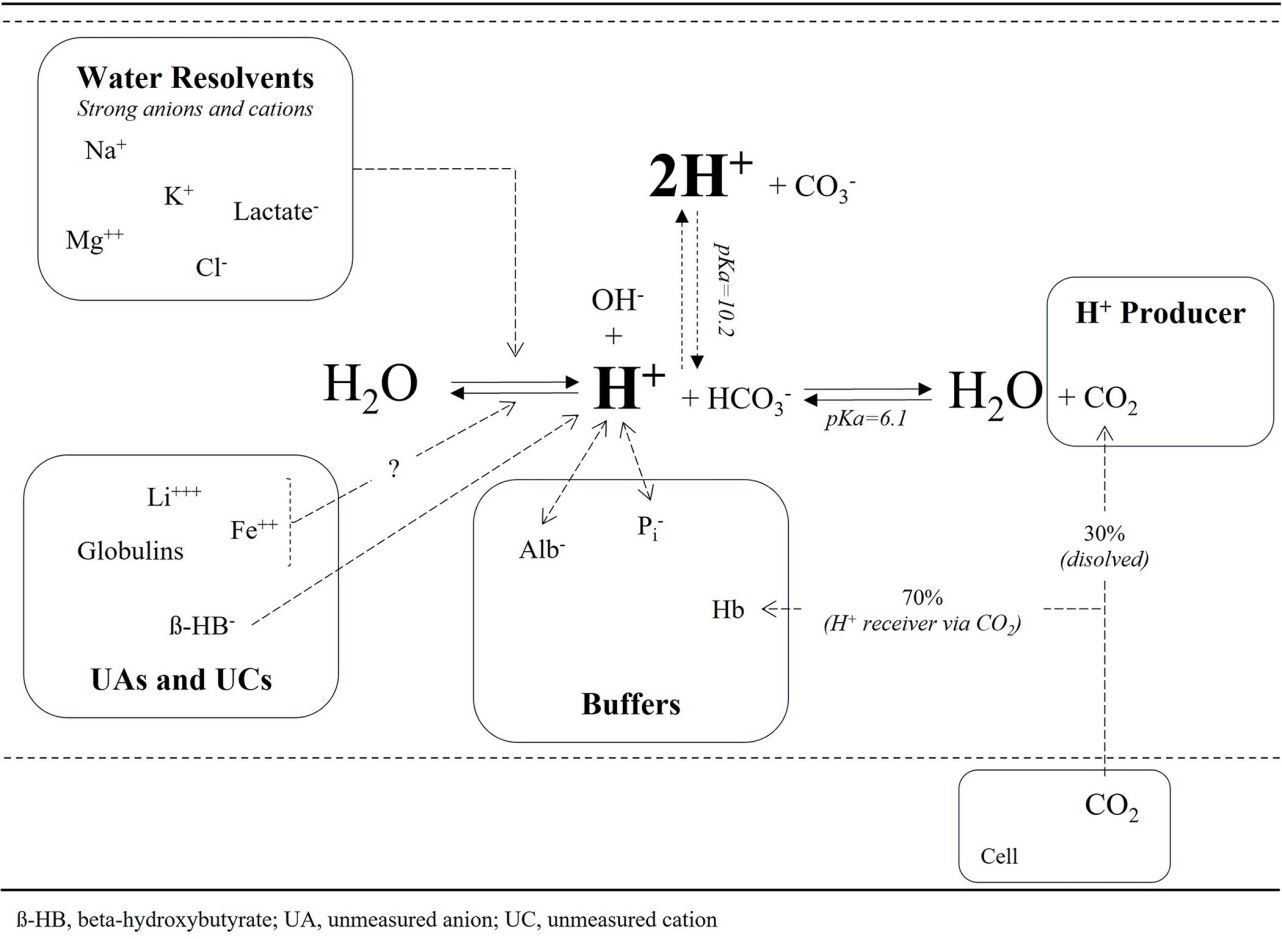
Effect mechanisms of independent variables on [H].

According to Stewart’s approach, the difference between strong ions affects water dissociation. Combining this knowledge with our results, we can conclude that deviations from normal serum values of each electrolyte and lactate affect water dissociation only (Fig 4). Among these, Na, Cl, and lactate ionic charges have substantial effects on [H] separately (Fig 2).

### Buffers (albumin and ionic phosphorus)

Albumin and P_i_ are named as weak acids in Stewart’s approach [7, 18]. They do not affect [H] on water dissociation but bind hydrogen ions when hydrogen ions increase in the plasma and vice versa (Fig 4). Indeed, these buffers’ ionic charges are affected by the pH in accordance with their ionic charge formula [12, 18], but at the same time, they are also independent variables of pH (Table 2). This indicates that even though albumin and Pi serum levels do not change, their ionic charges vary when pH increases or decreases. Another meaning is that albumin and P_i_ continuously correct the changes in pH due to the effects of other independent variables. Albumin may do this by changing its structure [26]. On the other hand, decreases and increases in albumin result in alkalosis and acidosis, respectively [9]. This situation can only be explained by the existence of an inverse relationship between albumin level and its affinity to hydrogen ions. Albumin’s affinity to hydrogen ions increases in hypoalbuminemia and decreases in hypoalbuminemia. Interestingly, this relationship is the exact opposite of that between hydrogen ions and hemoglobin, another essential plasma buffer. It seems that albumin works to protect the oxygen-binding capacity of hemoglobin because the most crucial duty of hemoglobin is oxygen transportation.

### Hydrogen ion producers (CO_2_: An indisputable independent variable of all approaches)

CO_2_ is neither a water resolvent nor a buffer in the plasma. It is only a hydrogen producer in the H_2_CO_3_ reaction (Fig 4). For this reason, it is an independent variable of pH and has the most important effect on it (Fig 2). However, this effect should involve the Hb buffer effect because CO_2_ transporting occurs via the same reaction in erythrocytes. On the other side, although HCO_3_ seems to be a buffer in this reaction, its buffer effect was not enough to make it an independent variable of pH in our results (when we added HCO_3_ to Model III, the p-value for HCO_3_ was 0.125). HCO_3_ and hydrogen ions are already end-products of this reaction and are associated with CO_2_. Therefore, the outlook on this reaction should be changed.

### Unmeasured anions (beta-hydroxybutyrate, D-lactate) and cations (Fe, Li, globulins)

Unmeasured anions/cations are undoubtedly important independent variables of pH (Table 2 and Fig 2). However, we still have to calculate their effect using the SIG formula, which may be a limitation [14, 18]. We believe that our new approach in Part II can logically solve this limitation. UA may have different mechanisms to affect [H]. Therefore, we have avoided categorizing their effect mechanisms (Fig 4).

The study’s most important limitation is that it is a single-center study. On the other hand, as new metabolic parameters are started to be measured routinely over time, these independent parameters may increase. Thus, this topic may be verified and improved by multicentric prospective studies in the future.

## Conclusions

pH has ten independent variables, and we reach the best prediction for pH when using all of them. Therefore, each of them should be separately evaluated when interpreting acid-base status. Each independent variable has an individual acid-base effect on top of its clinical importance. For this reason, it is unnecessary to create calculated parameters from them. On the basis of our results, we argue that blood gas machines have to measure each independent variable because it is their only purpose of existence. Evaluating these independent variables separately will yield more authentic results, rendering all complicated algorithms unnecessary.

## Supporting information

S1 Dataset (XLSX)

## References

[1] Siggaard-Andersen O. The Acid-base Status of the Blood. 1974. 13989038

[2] Singer RB, Hastings AB. AN IMPROVED CLINICAL METHOD FOR THE ESTIMATION OF DISTURBANCES OF THE ACID-BASE BALANCE OF HUMAN BLOOD. Medicine. 1948;27: 223. doi: 10.1097/00005792-194805000-00003 18868281

[3] The Great Trans-Atlantic Acid-Base Debate. Anesthesiology. 1965;26: 595–595. 14338914

[4] Oh MS, Carroll HJ. The anion gap. N Engl J Med. 1977;297: 814–817. doi: 10.1056/NEJM197710132971507 895822

[5] Siggaard-Andersen O. The van Slyke equation. Scand J Clin Lab Invest Suppl. 1977;146: 15–20. doi: 10.3109/00365517709098927 13478

[6] Gamble JL. Chemical Anatomy, Physiology, and Pathology of Extracellular Fluid. Fifth Edition. Soil Science. 1952. p. 403. doi: 10.1097/00010694-195211000-00009

[7] Stewart PA. Modern quantitative acid-base chemistry. Can J Physiol Pharmacol. 1983;61: 1444–1461. doi: 10.1139/y83-207 6423247

[8] Gabow PA, Kaehny WD, Fennessey PV, Goodman SI, Gross PA, Schrier RW. Diagnostic importance of an increased serum anion gap. N Engl J Med. 1980;303: 854–858. doi: 10.1056/NEJM198010093031505 6774247

[9] Fencl V, Leith DE. Stewart’s quantitative acid-base chemistry: applications in biology and medicine. Respir Physiol. 1993;91: 1–16. doi: 10.1016/0034-5687(93)90085-o 8441866

[10] Story DA, Poustie S, Bellomo R. Estimating unmeasured anions in critically ill patients: anion-gap, base-deficit, and strong-ion-gap. Anaesthesia. 2002;57: 1109–1114. doi: 10.1046/j.1365-2044.2002.02782_2.x 12428637

[11] O’Dell E, Tibby SM, Durward A, Aspell J, Murdoch IA. Validation of a method to partition the base deficit in meningococcal sepsis: a retrospective study. Crit Care. 2005;9: R464–70. doi: 10.1186/cc3760 16137362 PMC1269470

[12] Schück O, Matousovic K. Relation between pH and the strong ion difference (SID) in body fluids. Biomed Pap Med Fac Univ Palacky Olomouc Czech Repub. 2005;149: 69–73. 16170391

[13] Hatherill M, Waggie Z, Purves L, Reynolds L, Argent A. Correction of the anion gap for albumin in order to detect occult tissue anions in shock. Arch Dis Child. 2002;87: 526–529. doi: 10.1136/adc.87.6.526 12456555 PMC1755806

[14] Kellum JA, Kramer DJ, Pinsky MR. Strong ion gap: A methodology for exploring unexplained anions. Journal of Critical Care. 1995. pp. 51–55. doi: 10.1016/0883-9441(95)90016-0 7647842

[15] Albert RK, Slutsky A, Ranieri M, Torres A, Takala J. Clinical Critical Care Medicine. Mosby; 2006.

[16] Website. Available: gnesium Unit Conversion Page:: MediCalculator::: ScyMed::: [Internet]. [cited 2022 Jun 18];Available from: http://www.scymed.com/en/smnxps/psxrq261_c.htm

[17] Website. Available: ScyMed. MediCalc [Internet]. [cited 2022 Jun 18];Available from: http://www.scymed.com/en/smnxtb/tbcbktk1.htm

[18] Kellum JA, Elbers PWG. Stewart’s Textbook of Acid-Base. Lulu Press, Inc; 2013.

[19] Tucker AM, Johnson TN. Acid-base disorders: A primer for clinicians. Nutr Clin Pract. 2022;37: 980–989. doi: 10.1002/ncp.10881 35752932

[20] Hamilton PK, Morgan NA, Connolly GM, Maxwell AP. Understanding Acid-Base Disorders. Ulster Med J. 2017;86: 161–166. 29581626 PMC5849971

[21] Berend K. Diagnostic Use of Base Excess in Acid-Base Disorders. N Engl J Med. 2018;378: 1419–1428. doi: 10.1056/NEJMra1711860 29641969

[22] Fenves AZ, Emmett M. Approach to Patients With High Anion Gap Metabolic Acidosis: Core Curriculum 2021. Am J Kidney Dis. 2021;78: 590–600. doi: 10.1053/j.ajkd.2021.02.341 34400023

[23] Seifter JL, Chang H-Y. Disorders of Acid-Base Balance: New Perspectives. Kidney Dis (Basel). 2017;2: 170–186. doi: 10.1159/000453028 28232934 PMC5260542

[24] Hess DR, Kacmarek RM. Essentials of Mechanical Ventilation, Fourth Edition. McGraw Hill Professional; 2018.

[25] O’Dell E, Tibby SM, Durward A, Murdoch IA. Hyperchloremia is the dominant cause of metabolic acidosis in the postresuscitation phase of pediatric meningococcal sepsis. Crit Care Med. 2007;35: 2390–2394. doi: 10.1097/01.CCM.0000284588.17760.99 17717489

[26] Baler K, Martin OA, Carignano MA, Ameer GA, Vila JA, Szleifer I. Electrostatic unfolding and interactions of albumin driven by pH changes: a molecular dynamics study. J Phys Chem B. 2014;118: 921–930. doi: 10.1021/jp409936v 24393011 PMC3983335

